# Improving NGOs’ participation in implementing HIV preventive interventions: a case of adolescents with high-risk behaviors in Iran

**DOI:** 10.1186/s12889-025-21509-w

**Published:** 2025-02-07

**Authors:** Haniye Sadat Sajadi, Laleh Ghadirian, Azadeh Sayarifard, Fatemeh Rajabi, Maryam Nazari, Narges Rostamigooran, Nina Loori, Haniyeh Haji Abolhasan Memar, Mojgan Farshadi, Parvin Afsar Kazerooni, Maryam Sargolzaeemoghaddam, Reza Majdzadeh

**Affiliations:** 1 Knowledge Utilization Research Center, Tehran University of Medical Sciences, Tehran, Iran; 2 University Research and Development Center, Tehran University of Medical Sciences, Tehran, Iran; 3 Community Based Participatory Research Center, Tehran University of Medical Sciences, Tehran, Iran; 4 Shiraz HIV/AIDS Research Center, Shiraz University of Medical Sciences, Shiraz, Iran; 5 HIV/AIDS management in Center for Disease Control, Ministry of Health and Medical Education, Tehran, Iran; 6 School of Health and Social Care, University of Essex, Wivenhoe Park, Colchester, CO4 3SQ UK; 7 Center for Academic and Health Policy, Tehran University of Medical Sciences, Tehran, Iran

**Keywords:** Community participation, Non-governmental organizations, HIV, Acquired immunodeficiency syndrome, Adolescent, Health policy, Developing countries

## Abstract

**Background:**

The study aimed to identify the obstacles that NGOs face in their participation in implementing HIV preventive interventions among adolescents with high-risk behaviors in Iran and to propose interventions to enhance their involvement.

**Methods:**

The study employed a qualitative approach in three phases to identify barriers and solutions to NGOs’ participation in HIV preventive interventions. First, 56 semi-structured interviews, four focus group discussions (FGDs), and a document review were conducted with diverse stakeholders, using a purposive sampling strategy combining maximum variation sampling with a snowballing approach. Participants were sampled from relevant backgrounds in health policymaking, public participation, or communicable disease. Data from interviews and FGDs were audio-recorded, transcribed, and analyzed using an inductive content analysis. Second, a scoping review was performed, utilizing databases such as PubMed, Web of Science, ProQuest, and Google Scholar. A policy brief from the first two phases informed a one-day multi-stakeholder policy dialogue with 16 selected policy actors. This session was audio-recorded, transcribed, and analyzed through content analysis.

**Results:**

We identified various challenges faced by NGOs, including those related to the unique features of HIV services, such as difficulty in case finding and constant provision of preventive care. Severe challenges included weak NGOs’ performance, insufficient capabilities, and insufficient support from the government, resulting in undesirable constructive collaboration. Tailored strategies were developed, such as the empowerment of NGOs, enhancing public health literacy, modifying the process of identifying eligible NGOs, clarifying key processes for NGOs’ involvement, response to COVID-19, increasing adolescent engagement, advocating for removing the stigma from active NGOs, increasing support for active NGOs, organizing communication networks and collaboration, and strengthening governance arrangements.

**Conclusions:**

NGOs’ participation in HIV prevention can facilitate the alignment of interventions with the specific needs of at-risk populations. However, there are obstacles to full NGOs’ participation from both NGO and government perspectives, necessitating several measures to address these challenges. These measures are imperative for fostering constructive and sustained collaboration between NGOs and the government. The significance of this study lies in its emphasis on such collaboration, particularly in low-resource settings, which is crucial for nations committed to authentically realizing the goal of ‘health for all, by all,’ alongside genuine community participation.

**Trial registration:**

NA.

**Supplementary Information:**

The online version contains supplementary material available at 10.1186/s12889-025-21509-w.

## Background

Adolescents are regarded as one of society’s most susceptible groups to HIV infection due to several factors, such as physiological, psychological, and social problems. These can manifest in failed marriages, high-risk pregnancies, infertility, and sexually transmitted diseases [1]. In 2017, approximately 3.9 million adolescents aged 15–24 globally lived with HIV [2]. Without effective prevention and care interventions aimed at adolescents, experts project that new adolescent infections will potentially rise by up to 60% by 2030 [3].

It is widely accepted that prevention is the most optimal way to control HIV, given the absence of a definitive cure [4]. Preventive interventions for HIV/AIDS, based on World Health Organization’s guidelines [5], combine biomedical, behavioral, and structural approaches. Key measures include pre-exposure prophylaxis (PrEP), condom distribution, voluntary male circumcision, needle exchange programs, and antiretroviral therapy (ART) for prevention. These are supported by education, counseling, and efforts to reduce stigma and ensure equitable healthcare access. The non-governmental organizations (NGOs) have a crucial role in preventing HIV by imparting awareness through training workshops, disseminating HIV/AIDS-related information, and providing essential support services such as counseling to individuals living with HIV/AIDS [6–8]. Importantly, they can provide services efficiently to high-risk groups, including adolescents.

NGOs are generally non-profit, voluntary, and autonomous institutions recognized by governments, often focusing on broad-scale advocacy, policy-making, and service delivery across various issues contributing to human and social development [9, 10]. NGOs differ from community-based organizations (CBOs) in their scale and scope. While NGOs are typically larger, often operating at national or international levels, CBOs are smaller, locally focused groups that address specific community needs. CBOs are deeply rooted in the community, relying on local participation and leadership, which allows them to engage more directly in grassroots activities. Despite these differences, both NGOs and CBOs are forms of civil society organizations with a shared goal of addressing social issues, improving community well-being, and empowering individuals. They often collaborate to promote sustainable development and ensure equitable access to resources, working alongside governments and other key health stakeholders [11, 12].

Although NGOs have been effective in implementing HIV prevention measures and averting AIDS, their productive involvement in health promotion is sometimes impeded by different obstacles. Therefore, analyzing their strengths, weaknesses, and capacity is necessary to identify effective strategies and interventions in supporting NGOs. By pursuing these measures, NGOs can enhance social participation and expedite their progress towards enhancing the health and well-being of all [13].

Iran is significantly impacted by the global HIV epidemic, with an estimated 43,000 individuals (both adults and children) living with HIV in 2023, of whom 24,000 are aware of their HIV status [14]. Despite ongoing efforts, the rising incidence of HIV infections highlights the need for further interventions, particularly among high-risk groups such as adolescents [15, 16]. To address this, the country’s National HIV Strategic Plan integrates evidence-based biomedical, behavioral, and structural interventions aimed at reducing new infections among key populations. Key strategies include education to promote safer behaviors, the distribution of PrEP and condoms, harm reduction for people who inject drugs, STI care and treatment, HIV testing for early detection, strengthening surveillance and infrastructure, and enhancing healthcare infrastructure. Moreover, the plan places greater emphasis on the participation of NGOs, leveraging their capabilities to strengthen healthcare delivery. NGOs are tasked with delivering education targeted at high-risk groups, PrEP and condom use, and leading harm reduction efforts to ensure these interventions effectively and sustainably reach high-risk populations [17].

In keeping with several other countries, NGOs in Iran play an integral part in civil society and have a diverse range of activities to manage. Although the exact number of Iranian NGOs and their activities remain unclear, they are involved in various domains such as health, women’s issues, child welfare, environmental conservation, and education. Research highlights numerous challenges Iranian NGOs confront, including an arduous registration system, inadequate collaboration with other NGOs, socioeconomic sectors, governmental authorities, internationalization, and financial crises [18, 19]. Nevertheless, during the COVID-19 pandemic, NGOs have acted as an indispensable resource for Iranian society, and there is an emerging expectation that they will take on a greater role in upcoming health plans [20].

Given the alarming incidence of HIV contraction among Iranian adolescents [15, 16], their limited knowledge about HIV prevention (18.27%) [14], and the pressing need for greater involvement from NGOs in implementing HIV prevention interventions as outlined in the national strategic plan [17], it is essential to conduct a comprehensive analysis of the current situation of NGOs and develop effective improvement strategies. Although several studies in Iran have investigated the challenges of NGOs’ participation, their scope has generally been broad and not disease-specific. Consequently, there is a lack of research specifically addressing the role of NGOs in combating HIV. This study aims to identify the hurdles faced by NGOs in implementing HIV preventive interventions targeting high-risk behavior adolescents in Iran. Additionally, the study proposes feasible measures to enhance NGOs’ capacity for executing such interventions effectively.

## Methods

### Study design

**Fig. 1:**
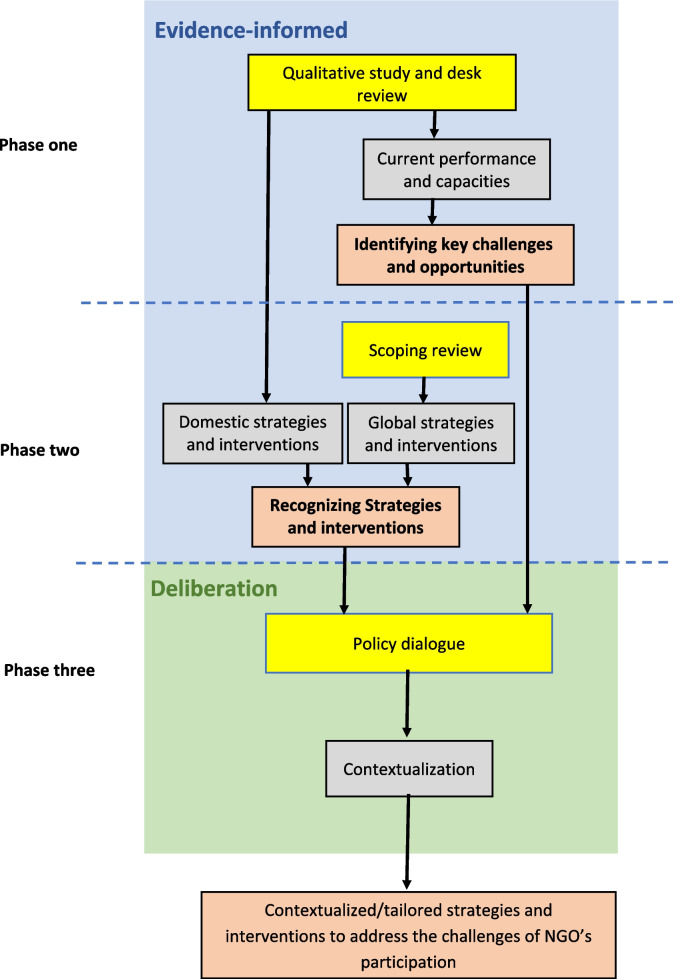
Sequence of the study phases with their methodology (yellow), subjects (gray) and output (orange)

From a constructivist point of view, we adopted an interpretative qualitative methodology [21] using the evidence-informed deliberative process [22] to pinpoint the main obstacles and interventions that could address the obstacles impeding NGOs’ participation. This approach aims to enhance their capacity to implement HIV preventive interventions targeted at high-risk behavior adolescents in Iran. The research comprised three distinct phases: interviews, document analysis, scoping review, and policy dialogue (refer to Fig. 1). Phase one identified NGOs’ key challenges and opportunities in implementing HIV preventive interventions. It was achieved through interviews, focus group discussions (FGDs), and document analysis, employing triangulation to ensure comprehensive and robust data gathering [23]. Phase two aimed to ensure evidence-informed policymaking by extracting and summarizing a list of potential interventions that could enhance NGOs’ participation in implementing preventive healthcare. A scoping review was conducted to find global strategies and interventions. The review has been published elsewhere [24]. At the end of these phases, a policy brief was developed, with a special focus on the challenges of NGOs in implementing HIV preventive interventions targeting high-risk behavior adolescents in Iran. Afterward, possible options to address the challenges were introduced. The policy brief was developed to spur the policy dialogue participants to be more active, help finalize the challenges, and contextualize the interventions [25]. Phase three involved a policy dialogue to examine the stakeholders’ perceptions and opinions about the barriers and solutions identified.

Below, we utilized the Standards for Reporting Qualitative Research (SRQR) checklist [21] to provide more detailed explanations of data gathering and analysis in this study.

### Reflexivity statement

In this study, all researchers are experienced in policy development and implementation within the context of middle-income countries. Among the researchers, one is male. Our professional backgrounds encompass communicable diseases, community participation, epidemiology, preventive medicine, and health policy and systems research. Two authors have been involved in national-level policymaking and believe addressing HIV issues requires greater community engagement. The remaining authors collectively believe that health interventions, particularly for hard-to-reach and vulnerable populations, should be designed through a scientific and evidence-informed approach and by engaging the target community, including empowering NGOs. While NGOs can effectively connect with the target community and facilitate identifying health needs and co-designing solutions, establishing a constructive collaboration with them can be challenging. However, it needs to be considered to accelerate the achievement of universal health coverage (UHC).

### Study setting

Iran is a country located in southwestern Asia. With a population of nearly 88 million people and an annual GDP growth of 5%, Iran is classified as a lower-middle-income country [26]. Iran has a relatively low HIV prevalence among the general population, with less than 0.1% of adults aged 15–49 living with HIV. However, certain high-risk groups face significantly higher rates of infection. The country has made strides in providing HIV prevention and treatment services, with over 18,596 people living with HIV receiving ART [27]. Despite these efforts, challenges remain, including societal stigma, limited funding, and the impact of economic sanctions, which affect the availability of resources and the sustainability of health programs. Addressing these issues is critical for improving the effectiveness of HIV interventions and ensuring better health outcomes for those at risk.

### Study participants

In this study, the participants were selected from individuals who (1) had an active and critical role in HIV policymaking at the national level, (2) were involved in providing HIV preventive interventions and implementing HIV programs throughout the country, or (3) were the main target groups of HIV prevention interventions and entitlement to receive HIV care. Among the eligible participants, only those willing to share their knowledge and expertise with the researchers were included in the study.

Based on these criteria, three groups of participants were identified for the interviews. Group I (policymaking level) included policymakers (*n* = 6) and managers from relevant national and international organizations (*n* = 3), specifically: one from the UNAIDS Iran Country Office (an international organization), one from the Welfare Organization and one from the Ministry of Sport and Youth (national governmental organizations). Group II (care providers level) comprised representatives from NGOs actively involved in delivering HIV preventive interventions nationwide (*n* = 20), healthcare providers engaged in HIV programs (*n* = 8), and managers of Adolescent Well-being Clubs (*n* = 7). It is worth noting that all NGOs involved in HIV preventive interventions were local at the time of the study. No international NGOs were working in the area of HIV services. Group III (caregivers level) consisted of adolescents (*n* = 10). For FGDs, the participants (*n* = 26) were selected among adolescents with high-risk behaviors who attended Adolescent Well-being Clubs since these clubs provided HIV prevention services to adolescents with high-risk behaviors (see Supplement 1 - Table A for more details of the study’s participants). For the policy dialogue, efforts were made to ensure that at least one representative from each group was present. For the session, invitations were sent to 20 key informants, of which 16 participants agreed to participate. The participants comprised six representatives from governmental organizations, eight from NGOs, and two adolescents. The gender distribution was relatively balanced, with approximately 44% being female, demonstrating the inclusivity of our study.

We were committed to ensuring our study captured the most diverse stakeholder perspectives. We employed a purposive sampling strategy that combined maximum variation sampling with a snowballing approach [28]. This approach allowed us to identify potential participants with varying backgrounds in health policymaking, infectious diseases, community participation, health management, and experience receiving HIV services. Our relatively heterogeneous study populations further enhanced our data richness, which led us to conduct 54 semi-structured in-depth interviews, four FGDs, and one policy dialogue. To ensure data saturation, we used the code frequency counts approach [29], which involves reviewing each interview or focus group transcript and counting the number of new codes in each successive transcript or set of transcripts until the frequency of new codes diminishes with few or no more codes identified.

### Document selection

Documents related to NGOs participation and HIV preventive programs were identified through (i) relevant departments and organizations website searches, including Ministry of Health and Medical Education (e.g., Center for Diseases Control, Bureau of AIDS and Sexually Transmitted Infections, HIV/AIDS Information Center), National Welfare Organization, Ministry of Cooperatives, Labour and Social Welfare (e.g., Office of Social Harms Control), Ministry of Sport and Youth, National AIDS Network, Ministry of Education, Ministry of Interior); (ii) google search; and (iii) expert consultation with key informants.

### Interview procedure

We performed qualitative, semi-structured interviews that lasted 30–90 min in October and November 2020. Three authors (LG, AS, and FR) with experience in qualitative research conducted one-to-one interviews. The interview guide was developed and utilized with minimal questioning. The guide had open questions on how NGOs have been actively involved in HIV preventive interventions for adolescents, exploring the challenges to NGOs’ collaborations, adolescents’ participations and identifying practical policy options, interventions, and solutions to improve their capacity for effective participation. Preliminary interviews were carried out as a pilot, and a slight amendment was made in the interview guide to ask to share the relevant documents or programs of success and failure of NGOs’ involvement in health provision and to say some real stories about their experience in providing HIV preventive interventions for adolescent. Considering the COVID-19 pandemic’s limitations, interviews were conducted over the phone after obtaining verbal informed consent from all interviewees. The interviews were audio recorded and transcribed verbatim. Summaries of the interviews were emailed to some participants for revision and completion if necessary.

### FGDs procedure

We employed FGDs with adolescents to allow us for further in-depth exploration of adolescents’ perspectives, experiences, and attitudes about the challenges of NGOs in implementing HIV preventive interventions targeting high-risk behavior groups. FGDs facilitate dynamic interactions and discussions that can reveal insights not easily captured through face-to-face interviews. This approach is useful for understanding complex issues, such as the barriers NGOs face in implementing HIV preventive interventions, as it encourages participants to share their thoughts, leading to a richer and more nuanced understanding of the topic. Four FGDs (two for girls and 2 for boys) were organized, with the participation of 12 girls and 14 boys. Two researchers (FR and MS) facilitated FGDs. The facilitators ensured that all participants had the opportunity to express their views. The main questions of FGDs were about how adolescents use the services provided, the factors affecting adolescent utilization and satisfaction, the level of adolescent participation, and the factors affecting it. FGDs were conducted via Skype and lasted between 1.5 and 2 h. They were held in November of 2020. In FGD‌ sessions, informed consent was obtained for participation after explaining the project’s objectives. With the permission of the teenagers, the discussions were recorded. We reassured them about the confidentiality of the discussions, and it was suggested that, if the adolescents so wished, the recording could be turned off or not transcribed in some parts of the discussion. The possibility of leaving the study at any stage was initially suggested. Taking notes was done during the meeting. The recorded topics were fully transcribed.

### Policy dialogue procedure

To ensure the dialogue was based on the most relevant knowledge, the policy brief prepared in the previous phases was distributed a week before the sessions. Thus, the participants were informed about the study’s objectives and the issues that should be discussed. The policy dialogue session lasted approximately 121 min, providing an extensive perspective of our research evidence and tacit knowledge of those involved in planning and providing HIV services. One of the authors (LG) facilitated the session. During these deliberative dialogues, we discussed the issue from different perspectives and agreed on the identified challenges. Secondly, a series of discussions were made around various solutions to resolve the problem and their feasibility. The session did not follow to reach a consensus, just looking to investigate different perspectives around the challenges and implementation considerations crucial to successfully implementing the proposed interventions. The session was held via Skype. The session was recorded, and the data was transcribed with the consent of the participants.

### Data analysis

The data analysis was carried out using manifest content analysis [30] by four independent authors (HSS, LG, AS, FR). We meticulously adhered to the informants’ words, focusing on the visible and explicit elements in the text. Any disagreements were resolved through discussion. Coding was conducted using inductive and deductive approaches, and MAXQDA12 software facilitated the organization of results. This method is particularly effective when the research topic is well-known but limited available information exists.

Furthermore, quality criteria were considered, including credibility, transferability, dependability, and confirmability of qualitative studies [31]. To ensure credibility, researchers made trust with all participants. A description of the context and experiences of participants was utilized for transferability, while audit trails were used to guarantee dependability and confirmability. The documents and transcripts that were included were originally in Persian. The code descriptions, extracted from the interview transcripts and documents, were subsequently translated from Persian into English.

### Data extraction

One author (HSS) reviewed and extracted data from relevant documents, including nine formal reports and programs, ten guidelines and instructions, and nine regulations (see Supplement 1 -Table B for more details of the included documents). Information such as the title, type, year, the role of NGOs in health service provision, the challenges of their involvement, and opportunities to enhance their capacities were extracted and entered into a data extraction matrix developed in Microsoft Excel.

### Ethical consideration

In our study, we obtained informed oral consent from all participants and the parents of adolescents, highlighting their voluntary involvement. Before collecting data, we clearly explained the study’s purpose, procedures, potential risks, and benefits. Participants were thoroughly informed and allowed to ask questions or seek clarification. To protect their privacy, we strictly upheld participant confidentiality. During data analysis and reporting, we carefully removed any identifying information.

## Results

In phase one of our study, we analyzed the challenges faced by NGOs in their participation in HIV preventive interventions targeted at high-risk behavior adolescents in Iran. Our data analysis generated 279 codes, which were then categorized into 11 thematic challenges and 40 sub-themes, as shown in Table 1. Similarly, we identified 232 codes for possible solutions aimed at improving the participation of NGOs in HIV preventive interventions for high-risk behavior adolescents in Iran. These solutions were then classified into three categories (levels), 14 themes (strategies), and 52 sub-themes (interventions), as demonstrated in Table 2. These strategies and interventions mainly covered the managerial strategies and executive requirements to strengthen the participation of NGOs in implementing HIV preventive care interventions in adolescents in Iran. They also covered different levels of improvement, including individual, organizational, and system levels.

In phase two of our study, we identified 31 interventions and categorized them into 11 strategies. These strategies included building strong collaborations among NGOs and with governments, expanding networks and sustaining relationships among NGOs, evaluating the performance of NGOs, increasing intersectoral collaboration, advocating for the role of NGOs, supporting NGOs from the side of government, empowering the abilities and capabilities of NGOs, defining the precise roles and responsibilities of the parties, strengthening the health system governance, increasing the health literacy of the community, and developing required regulations, rules, and policies. Unfortunately, none of the interventions identified had evidence of their effectiveness [24].

In phase three of our study, we did not identify any new challenges, strategies, or interventions for enhancing NGOs’ participation in HIV prevention efforts for adolescents with high-risk behaviors in Iran (Table 3). For each challenge (column 1), we specify one or more strategies (column 2) based on the policy dialogue. This problem-based approach ensures that at least one strategy is listed for each challenge. The table also indicates which strategies are supported by global evidence from a scoping review (column 4). As shown, 10 out of 14 identified strategies are supported by global evidence. To contextualize, we assess each strategy’s perceived effectiveness (column 5) and feasibility (column 6) based on the dialogue. More stars indicate higher perceived effectiveness and feasibility. The final column presents the main implementation considerations discussed during the policy dialogue.

**Table 1:** The challenges for NGOs’ participation in implementing HIV preventive interventions in adolescents with high-risk behaviors in Iran

Challenge	Sub-Challenge
Unique features of HIV services ‌‌‌	High HIV exposure and prevalence in lower ages ‌‌
	Necessity of taking quick and persistent preventive measures ‌
	Extent of HIV services ‌‌
Difficulty in identifying, engaging, communicating, and providing ongoing services to high-risk adolescents	Adolescents’ tendency towards high-risk behaviors and exposure to high-risk environments ‌ ‌‌
	Lack of adolescents’ tendency towards HIV preventive services among high-risk adolescents
	Adolescents’ special temperaments and the difficulty of earning their trust and effective engagement
	Difficulty of access to adolescents, especially in vulnerable and special groups
Common social and family limitations in dealing with HIV ‌	Low health literacy level of families about HIV
	Inadequate cooperation from parents and families ‌
	Risk factors in the family environment ‌
	Cultural and security contexts governing HIV services in society ‌‌
Weak communication networks and organizational interactions with high-risk adolescents in organizations in the field of HIV preventive services ‌‌‌‌	Lack of knowledge of related organizations of cooperation capacities ‌‌
	Insufficient intersectoral collaborations among related organizations ‌
	Poor intra-organizational coordination in related organizations ‌‌
Lack or inadequacy of clear laws and regulations on the mechanism of NGOs’ involvement and dissemination of information ‌‌	Defects in the protocol of adolescent health clubs‌‌
	Lack of necessary laws and instructions or defective laws and instructions ‌
	Lack of knowledge of some NGOs of existing laws ‌
Poor performance assessment over involvement of NGOs (including clarity of services, evaluation criteria, evaluation team, and mechanism of using results) ‌‌	Lack of performance assessment indicators and tools over the NGOs’ functioning ‌‌
	Limitation of required resources for performance assessment
	Unclear supervisory role of different organizations
	Uncertainty about the mechanism of using performance assessment results
Unspecified process of service delivery with the cooperation of NGOs	Ignoring geographical differences in service delivery process (time and method of performing activities) ‌
	Uncertainty about services that NGOs can provide ‌
	lack of clarity on NGOs’ cooperation details ‌
Unspecified position and role of NGOs’ cooperation in service delivery ‌	Lack of knowledge and information of the public and governmental organizations about the role of NGOs ‌‌
	Negative perception toward NGOs’ activities ‌
	Poor communications between NGOs and organizations related to HIV prevention ‌‌‌‌
	Managerial changes and lack of adequate knowledge and commitment of managers and experts to NGOs’ activities ‌
Shortage of NGOs with adequate capacity for cooperation ‌‌	Shortage of active NGOs ‌
	Features governing the formation and activities of NGOs ‌‌
	Unclear mechanism for selecting partner NGOs ‌
Limitations of NGOs during the execution of the program ‌‌	The inability of some NGOs to supply, distribute, and allocate financial resources ‌‌
	Shortage of competent human resources in NGOs for service delivery ‌‌
	The inability of some NGOs to supply necessary space and equipment for service delivery ‌
	Unfamiliarity with NGOs with the capacity to coordinate and interact with the public sectors and society ‌‌‌
	Lack of strong communication and social network between NGOs ‌
	Poor research capacity of NGOs
	COVID-19 pandemic and its consequence on NGOs’ service delivery ‌
Lack of sufficient support for NGOs and their activities from governmental organizations ‌	Limited human resources in governmental organizations to support NGOs ‌
	Weakness of governance structure at national and provincial levels

**Table 2:** The solutions to improve NGOs’ participation in implementing HIV preventive interventions in adolescents with high-risk behaviors in Iran

Level	Strategy	Intervention
Individual	1. Empowerment of active NGOs ‌	1.1. Holding a briefing program for selected NGOs ‌
		1.2. Periodic educational need assessment of human resources working in active NGOs ‌‌
		1.3. Holding general education courses on human resources for NGOs ‌‌
		1.4. Holding specialized education courses on human resources for NGOs
	2. Enhancing public health literacy and awareness	2.1. Raising public awareness about HIV and how to prevent it in high-risk adolescents and how to manage and eliminate HIV-related stigma through the mass media ‌‌‌‌‌‌
		2.2. Providing HIV prevention education to adolescents in schools ‌‌‌
		2.3. Providing HIV prevention education to parents (in religious schools, mosques, and similar centers in different institutions) ‌
Organization	3. Modifying the process of identifying and selecting eligible NGOs	3.1. Defining NGO selection criteria for their participation in the implementation of HIV preventive interventions in adolescents with high-risk behavior and disseminating related information to the public ‌‌‌‌‌
		3.2. Preparing, updating, and informing the list of active and interested NGOs ‌‌‌‌
		3.3. Periodic assessment of NGOs’ capacities (human, physical, and financial) ‌‌‌‌
	4. Clarifying the process of partnership with NGOs given geographical and urban differences	4.1. Developing and informing the list of services that NGOs can provide for implementing HIV preventive interventions in adolescents with high-risk behaviors
		4.2. Developing a memorandum of understanding the partnership between NGO and relevant organizations according to existing capacities for cooperation ‌‌‌
	5. Clarifying the process of performance assessment of active NGOs‌	5.1. Developing supervision criteria over activities of NGOs based on their assigned tasks in the implementation of HIV preventive interventions in adolescents with high-risk behavior ‌‌‌‌
		5.2. Determination of performance assessment mechanism (evaluators, evaluation method, and evaluation time) of NGOs’ activities based on assigned tasks in the implementation of HIV preventive interventions in adolescents with high-risk behaviors ‌‌‌‌
		5.3. Determination of the mechanism of using performance assessment results and financial or non-financial rewards ‌
	6. Facilitation of communications and interactions among NGOs	6.1. Strengthening the network of active NGOs in the implementation of HIV preventive interventions in adolescents with high-risk behavior (SHAMSA) ‌‌‌
		6.2. Providing a platform for the exchange of experiences (including clubs, NGOs, and related areas in the university and the ministry) ‌‌‌‌
		6.3. Forming a coalition of NGOs to participate in the implementation of HIV preventive interventions in adolescents with high-risk behaviors ‌‌‌
		6.4. Holding synergistic meetings, seminars, etc., to exchange views and experiences ‌‌
		6.5. Extracting, propagating, and using experiences of successful countries, especially Muslim countries, in engaging NGOs in the implementation of HIV preventive interventions in adolescents with high-risk behaviors ‌‌‌‌
	7. Effective response to COVID-19	7.1. Reviewing service delivery methods by NGOs according to consequences of the COVID-19 pandemic and with emphasis on the use of remote health services ‌‌
	8. Increasing meaningful adolescent engagement in all phases of planning and implementing the HIV preventive services	8.1. Holding a synergistic session with high-risk adolescents
		8.2. Active participation of adolescents in NGOs’ planning and service delivery to adolescents ‌‌
		8.3. Delegating authorities and responsibilities to adolescents ‌
		8.4. Holding attractive programs to persuade individuals to visit NGOs ‌‌
		8.5. Using peer groups and outreach teams
		8.6. Preparing appropriate educational packages for adolescents ‌
	9. Advocating and support for removing the stigma from active NGOs	9.1. Introducing NGOs and their roles in HIV preventive service delivery among high-risk adolescents to national policymakers and local authorities ‌‌‌‌
		9.2. Introducing NGOs and their roles in HIV preventive service delivery among high-risk adolescents to governmental organizations (administrative offices affiliated to different ministries, universities, schools, and other organizations) or social marketing for NGOs ‌‌‌‌‌‌
		9.3. Introducing NGOs and their roles in HIV preventive service delivery among high-risk adolescents to the public (families, civil organizations, religious leaders, and influences) ‌‌‌‌
		9.4. Introducing NGOs and their roles in HIV preventive service delivery among high-risk adolescents ‌‌‌
		9.5. Introducing SHAMSA and its activities to relevant governmental organizations ‌‌‌
		9.6. Setting HIV-related services among priorities of university donors’ office ‌‌
	10. Organizing communication networks and inter-organizational interactions in related organizations	10.1. Holding educational courses for experts in governmental organizations
System	11. More support of active NGOs by governmental organizations	11.1. Supplying necessary human resources specialized in the field of HIV in relevant institutions according to the number of active agents ‌
		11.2. Participating in supplying necessary physical spaces and equipment for service delivery by NGOs ‌
		11.3. Attracting help and financial support for active NGOs
		11.4. Providing specialized personnel for training NGOs ‌
		11.5. Supporting the presence of lecturers, students, and volunteers in NGOs ‌
	12. Organizing communication networks and intersectoral collaboration	12.1. Identifying key stakeholders in the field of NGOs’ involvement in the implementation of HIV preventive interventions among adolescents with high-risk behaviors ‌‌‌‌
		12.2. Clarifying the role and task of each stakeholder in interaction with active NGOs in the implementation of HIV preventive interventions among high-risk adolescents ‌‌‌‌‌
		12.3. The increasing number of sessions with the participation of representatives of relevant organizations ‌
	13. Developing, reviewing, and giving information about laws and regulations required for activities of NGOs	13.1. Reviewing the protocol of health clubs by considering regional conditions, the necessity of categorizing adolescents, paying attention to healthy adolescents and vulnerable populations (e.g., immigrants/refugees), and infection at lower ages ‌‌‌‌
		13.2. Developing ethical standards guidelines and adhering to confidentiality principles for adolescents to participate in the implementation of HIV preventive interventions
		13.3. Developing protocol of legal and judicial standards on activities of NGOs in the implementation of HIV preventive interventions ‌
		13.4. Developing a specific referral protocol (from other organizations to NGOs and vice versa) ‌
		13.5. Developing comprehensive guidelines for social harms ‌
		13.6. Unification of health NGOs licensing authorities
		13.7. Developing a complete educational content for HIV prevention in adolescents ‌‌
		13.8. Providing up-to-date information on instructions related to NGOs’ activities concerning their involvement in the implementation of HIV preventive interventions in high-risk adolescents ‌‌‌‌‌‌
	14. Strengthening the governance structure	14.1. Increasing activity of the Supreme Council of the SIP Committee at the national level to approve developed programs and guidelines and supervise their implementation ‌‌‌
		14.2. Operationalizing provincial and county committees of the SIP Committee at sub-national to supervise the implementation of programs ‌‌

**Table 3:** The capacity development plan for NGOs’ participation in implementing HIV preventive interventions in adolescents with high-risk behaviors in Iran

Challenge	Strategy	Level	Global evidence	Perceived effectiveness^a^	Feasibility	Implementation consideration
Unique features of HIV services	12. Organizing communication networks and intersectoral collaboration	Individual	α	**	***	
	13. Developing, reviewing, and giving information about laws and regulations required for activities of NGOs	Organization	α	**	***	- The experiences of Adolescent Well-being Clubs and NGOs should be used in developing protocols - It is essential to pay attention to the needs of adolescents, the nature and sensitivity of the issue (HIV), and legal considerations for NGOs as nongovernmental organizations when developing the protocols - The authority for the approval of the protocol is the SIP Committee at the national level - The protocol should be developed with the cooperation of the stakeholders - Given the sensitivity of the issue, key educational subjects should be added while the governing ethical principles are observed
Difficulty in identifying, engaging, communicating, and providing ongoing services to high-risk adolescents	1. Empowerment of active NGOs ‌	Individual	α	***	***	- Given the COVID-19 pandemic, these sessions can be held online - The skills required for NGOs’ activities should be prepared, updated, and available - Skills required for each NGO are determined based on the Memorandum of Cooperation - Given the COVID-19 pandemic, need assessment can be performed virtually Holding every educational course requires a need assessment - TOT training is recommended
	8. Increasing meaningful adolescent engagement in all phases of planning and implementing the HIV preventive services	Organization		**	**	
Common social and family limitations in dealing with HIV	2. Enhancing public health literacy and awareness	System	α	**	***	
	9. Advocating and support for removing the stigma from active NGOs		α	***	**	- The audience’s language should be considered when preparing introduction programs - The use of reputable NGOs can be effective in the introduction process ‌‌‌- The capacities of healthcare communicators can be used - Using common means of information dissemination, including television and Instagram, is recommended - The use of reputable NGOs can be effective in the introduction process ‌‌‌
Weak communication networks and organizational interactions with high-risk adolescents in relevant organizations	10. Organizing communication networks and inter-organizational interactions in related organizations	Organization		**	***	
	12. Organizing communication networks and intersectoral collaboration in related organizations	System	α	**	***	
Lack or inadequacy of clear laws and regulations on the mechanism of NGOs’ involvement and dissemination of information	6. Facilitation of communications and interactions among NGOs	Organization	α	**	***	
	13. Developing, reviewing, and giving information about laws and regulations required for activities of NGOs	System	α	**	***	
Poor performance assessment of the involvement of NGOs	5. Clarifying the process of performance assessment of active NGOs‌	Organization	α	***	***	- Besides the quantity, the service quality should also be considered in supervision criteria - The assessment criteria for appropriate NGO’s functioning are selected based on the memorandum of cooperation - Coordination with NGO licensing authority is essential to facilitate the performance assessment process - The evaluation team should be composed of representatives from the university, club, or the Supervising of the Implementation of the Program (SIP) Committee at the national level for HIV Prevention - The use and announcement of supervision results should not cause unhealthy competition between companies - The performance of each NGO is better compared to its status in the past or the status of similar NGOs
Unspecified process of service delivery with the cooperation of NGOs, given geographical and urban differences	4. Clarifying the process of partnership with NGOs given geographical and urban differences	Organization	α	**	**	- Generalities related to the memorandum’s conclusion should be prepared, and the partnership details (including the type of services, mechanism and time of service delivery, and supervision) should be specified based on geographical and urban areas - The protocol should be developed with the cooperation of both parties
Unspecified position and role of NGOs’ cooperation in service delivery	9. Advocating and support for removing the stigma from active NGOs	Organization	α	***	**	- The audience’s language should be considered when preparing introduction programs ‌ - The use of reputable NGOs can be effective in the introduction process ‌‌ ‌‌‌- The capacities of healthcare communicators can be used - Using common means of information dissemination, including television and Instagram, is recommended - The use of reputable NGOs can be effective in the introduction process ‌‌‌
Shortage of NGOs with adequate capacity for cooperation ‌‌	3. Modifying the process of identifying and selecting eligible NGOs	Organization		***	***	- Dissemination of information is recommended to be in the form of a general calling - NGO selection stages should be transparent - The number of active NGOs in each region should be increased while gender equality is considered - Capacity assessment criteria can be designed based on the existing protocol for health clubs
Limitations of NGOs during the execution of the program	1. Empowerment of active NGOs	Individual	α	***	***	
	6. Facilitation of communications and interactions among NGOs	Organization	α	**	***	
	7. Effective response to COVID-19			**	**	
	11. More support of active NGOs by governmental organizations	System	α	***	***	- An example of this support is the allocation of a place for the implementation of programs ‌ - An example of this support is providing free services to NGOs - Sustainability should be considered when providing resources - These training programs can be provided to NGOs free of charge ‌‌‌
Lack of sufficient support for NGOs and their activities from governmental organizations	11. More support of active NGOs by governmental organizations	System	α	***	***	
	14. Strengthening the governance structure		α	**	*	- The presence of NGOs and SHAMSA’s representatives in meetings of the SIP Committee is recommended ‌

^a^The stars indicate the perceived effectiveness and feasibility based on the policy dialogue discussion. More stars represent higher perceived effectiveness or feasibility

## Discussion

Through a problem-oriented methodology and stakeholder engagement, the study highlighted challenges such as difficulty in case finding, ongoing preventive care, weak NGOs’ performance, insufficient capabilities, and limited government support, which hindered effective collaboration. To overcome these barriers, tailored strategies were proposed, including empowering NGOs, improving public health literacy, refining NGO selection processes, clarifying involvement procedures, addressing COVID-19 impacts, engaging adolescents, reducing stigma, increasing support, fostering communication and collaboration networks, and strengthening governance arrangements.

The current investigation emphasizes an array of barriers that NGOs face while implementing HIV-preventative interventions. In line with prior research [32–34], certain hindrances are predominantly associated with the unique attributes of HIV disease and its modes of transmission and the cultural norms and values surrounding the ailment. These difficulties not only obstruct the endeavors of NGOs but also hinder the government’s delivery of critical services to patients. It is crucial to create and implement awareness-raising initiatives that focus on various sectors of society to tackle these problems, including religious leaders, and integrate various forms of information dissemination, such as films and messages [35–37]. One of the challenges identified is NGOs’ lack of participation in health services provision. It can be attributed to their weak capacity and the ambiguity surrounding their status and role in healthcare provision. This issue has been extensively documented in various studies and reports [18, 38], particularly in low- and middle-income couriers (LMICs) [39, 40].

However, the primary challenge appears to be the absence of a constructive relationship or interaction with government and public authorities, such as the Ministry of Health. However, previous studies [24] have emphasized that creating and sustaining a constructive and two-way interaction between NGOs and the government is a crucial prerequisite for the successful involvement of NGOs in community health participation.

As a consequence of these obstacles, the capacity of NGOs to advance HIV/AIDS preventive initiatives in Iran is not being fully exploited. It is despite NGOs’ recognized effectiveness in executing health interventions and advancing AIDS prevention, as demonstrated by prior research [6, 41]. Additionally, NGOs have been recognized for their capacity to engage with vulnerable and hard-to-reach populations [38, 42].

Our study suggests that three intervention groups are important to address NGOs’ challenges. The first group is centered on empowering the NGOs by enhancing their general skills (e.g., resource mobilization, planning, seeking support) and specific capabilities (e.g., knowledge of HIV/AIDS, building rapport with patients, diagnosing the disease). NGOs must continually upgrade their skills and knowledge to function more effectively like any organization. Enabling NGOs through a formalized training program is crucial for enhancing their involvement in implementing HIV/AIDS prevention initiatives and achieving health objectives [43, 44].

The second group of interventions is related to redefining the role and position of NGOs in health promotion programs and revising the different processes of NGOs’ work [45]. Determining the authorities and duties of NGOs in HIV/AIDS prevention measures, introducing NGOs, showing the positive effects of their performance in promoting health, and facilitating the exchange of experience and information are the main actions of this group of programs.

The third group of interventions focuses on enhancing intersectoral collaboration and involves government and government agencies actively supporting NGOs by establishing bilateral cooperation. These interventions have been studied extensively in the past [18, 24] and aim to foster constructive interaction between NGOs and the government, thereby enabling the government to leverage the potential of NGOs in achieving health goals. Such interventions may involve partnerships between NGOs and government agencies, joint projects aimed at delivering health services and interventions, or collaborations to address specific health challenges. These collaborations have the capacity to create synergies and maximize the impact of HIV/AIDS prevention initiatives, leading to improved health outcomes for communities.

The government must prioritize certain measures to establish a constructive interaction between NGOs and the government [12, 19, 24, 38]. One such measure is to provide a legal environment that supports the activities of NGOs. It can be achieved by formulating uniform and coordinated laws that regulate NGOs’ health sector activities. It will help ensure that NGOs operate within a clear legal framework with a well-defined scope of activities [24]. Another important measure is to provide technical support to NGOs’ activities in a way that minimizes bureaucracy and cumbersome procedures [19, 24]. It can involve providing training, capacity building, and access to resources such as financial support and information technology. Technical support will enable NGOs to carry out their activities more effectively, leading to better health outcomes. It is also important for the government to have constructive and effective supervision of NGOs’ activities [24, 38]. It involves monitoring and evaluating the impact of NGOs’ activities and providing feedback to ensure that the intended outcomes are achieved. It is important to strike a balance between ensuring accountability and transparency while also allowing NGOs the necessary autonomy to perform their activities.

Our study had both strengths and limitations. One of the significant strengths was involving all stakeholders in the process. The study aimed to seek opinions from different stakeholders, including policymakers, service providers, seniors, and teenagers, to identify challenges and propose solutions. It helped develop the final interventions and capacity-building program by soliciting opinions from all groups, making them familiar with the proposed program from the first stages of development. It increases the possibility of their participation in the implementation of the program. Another strength of the study was using a problem-solving approach to identify the interventions and present the capacity-building program. As highlighted in the last table, solutions to each challenge were specified, making a menu of policies and actions available to the policymaker for each challenge. It allows the policy maker to choose among them according to their resources and prioritization method. The study also attempted to use an evidence-informed approach to problem-solving. It recommended identifying local problems and using global evidence and national knowledge to develop solutions. As the study’s findings indicate, many of the interventions recommended to increase the participation of NGOs were identified through the qualitative study. Thus, this study provides evidence-informed solutions, increasing confidence in the effectiveness of these interventions.

Our study is not without limitations. First, limited evidence supports the effectiveness of the interventions identified in the review. While some of these interventions have previous evidence, we cannot deem them definitively effective due to the lack of strong evidence to support their effectiveness. The policy dialogue has also evaluated the evidence for the perceived effectiveness and implementation of these interventions. Therefore, although these interventions may be suitable for countries with a similar background to Iran, it is necessary to exercise caution when using the results.

Furthermore, more studies are required to confirm the effectiveness of these interventions. The second limitation of the study is that it focuses on local NGOs with a national scope. While, based on national regulations, all local and international NGOs must pass formal procedures to be allowed to work in Iran and collaborate in healthcare provision, the challenges faced by international NGOs may differ from those of local NGOs. When using the findings of this study, it is important to consider this limitation. However, it is important to emphasize that strengthening NGOs and leveraging their capacity—whether national or international—is crucial for LMICs facing limited resources. Given their contextual circumastance, strategies for strengthening NGOs should ensure that their role is aligned with and coordinated by national programs, while also guaranteeing the sustainability of resource provision through these organizations. In many LMICs, national NGOs are often most effective in delivering services and empowering communities, whereas international NGOs tend to play a more prominent role in providing technical assistance.

As highlighted in Daneshmand et al. [46], during severe crises, such as sanctions, governments often prioritize policies that enhance social cohesion and leverage locally-rooted solutions over reliance on external actors. Based on authors observations, during the presidency of Mahmoud Ahmadinejad, amidst heightened international disputes, the administration emphasized fostering internal cohesion. This approach coincided with the “Color Revolutions” in newly independent states of the former Soviet Union, where international NGOs were perceived to play significant roles. In this sensitive context, traditional civil society organizations, such as charitable foundations, increasingly gained prominence due to their alignment with national and cultural frameworks. This historical context underscores the importance of understanding how political realities shape the role and visibility of NGOs in such environments.

The third limitation is the absence of a monitoring and evaluation framework for the proposed interventions. Due to time and resource constraints, the proposed interventions lack a proper monitoring and evaluation framework. Thus, researchers are encouraged to conduct further studies to develop a monitoring and evaluation framework to assist in implementing these interventions. It will enable systematic tracking of the progress of these interventions and facilitate adjustments in strategies and activities where necessary.

## Conclusions

Our study identified several interventions to enhance NGOs’ participation in implementing HIV preventive measures. Our findings underscore the urgent need for strategic actions to strengthen NGOs’ role in delivering HIV services. Policymakers should prioritize NGOs’ empowerment by providing targeted training and resources to enhance their capabilities, thereby enabling them to address the unique challenges associated with HIV case finding and preventive care. Furthermore, increasing government support is essential for fostering constructive collaboration between NGOs and public health authorities.

Policies should also focus on enhancing public health literacy to better inform communities about HIV prevention and treatment while actively engaging adolescents in these initiatives. The importance of public health literacy cannot be overstated, as it is a key factor in effective community education about HIV prevention. Establishing clear processes for NGO involvement, improving communication networks, and strengthening governance arrangements will facilitate better coordination and resource allocation, ultimately increasing community engagement and achieving UHC [47]. Additionally, there is a pressing need for an organized approach to implement measures that strengthen NGO participation. National programs should be translated into operational plans, with their implementation subject to periodic monitoring and evaluation. Unfortunately, monitoring and evaluation are often neglected within the health systems of many LMICs [48]. Ensuring effective oversight is crucial, yet there remains a significant gap in program implementation and health system reforms.

## Supplementary Information

Supplementary Material 1

## Data Availability

The datasets used and/or analyzed during the current study are available from the corresponding author upon reasonable request.

## Abbreviations

ART: Antiretroviral therapy
CBO: Community based organization
FGD: Focus group discussion
GDP: Gross domestic product
LMICs: Low- and middle-income couriers
NGO: Nongovernmental organization
PrEP: Pre-exposure prophylaxis
SRQR: Standards for reporting qualitative research
UHC: Universal health coverage
