# Correction: Improving NGOs’ participation in implementing HIV preventive interventions: a case of adolescents with high-risk behaviors in Iran

**DOI:** 10.1186/s12889-025-24802-w

**Published:** 2025-11-03

**Authors:** Haniye Sadat Sajadi, Laleh Ghadirian, Azadeh Sayarifard, Fatemeh Rajabi, Maryam Nazari, Narges Rostamigooran, Nina Loori, Haniyeh Haji Abolhasan Memar, Mojgan Farshadi, Parvin Afsar Kazerooni, Maryam Sargolzaeemoghaddam, Reza Majdzadeh

**Affiliations:** 1 Knowledge Utilization Research Center, Tehran University of Medical Sciences, Tehran, Iran; 2 University Research and Development Center, Tehran University of Medical Sciences, Tehran, Iran; 3 Community Based Participatory Research Center, Tehran University of Medical Sciences, Tehran, Iran; 4 Shiraz HIV/AIDS Research Center, Shiraz University of Medical Sciences, Shiraz, Iran; 5 HIV/AIDS Management in Center for Disease Control, Ministry of Health and Medical Education, Tehran, Iran; 6 School of Health and Social Care, University of Essex, Wivenhoe Park, Colchester, CO4 3SQ UK; 7 Center for Academic and Health Policy, Tehran University of Medical Sciences, Tehran, Iran

**Correction: BMC Public Health 25, 520 (2025)**

**https://doi.org/10.1186/s12889-025-21509-w**

Following publication of the original article [1], the authors reported an error found in Ethics approval and consent to participate section.

The updated Ethics approval and consent to participate is given below and the changes have been highlighted in **bold typeface**.

**Ethics approval and consent to participate**

The approval for this study was granted by the Research Ethics Committees of **School of Medicine, Tehran University of Medical Sciences and Health Services (IR.TUMS.MEDICINE.REC.1399.807).**

The original article [1] has been updated.
