# Supplementary material for: Improving NGOs’ participation in implementing HIV preventive interventions: a case of adolescents with high-risk behaviors in Iran
Source: BMC Public Health. 2025 Feb 7;25:520. doi: 10.1186/s12889-025-21509-w (PMC11806659; doi:10.1186/s12889-025-21509-w)
Supplement: Supplementary file 1 — Supplementary Material 1 [file 12889_2025_21509_MOESM1_ESM.docx]

**Supplement 1-: Characteristics of study participants and relevant documents**

Table A- The study participants

| **Variable** | **Interviews** | | | **Focus group discussion** |
| --- | --- | --- | --- | --- |
|  | Group I: policymaking level (=9) | Group II: care providers level  (=35) | Group III: caregivers level (=10) |  |
| Gender  Female  Male  Age  <20  21-30  31-40  41-50  51-60  >60  Not mentioned  Education  PhD  MD  MS/MA  BS/BA  Diploma/high school  Not mentioned  Experience  <10  11-20  21-30  >30  Position  Managers  Officer/expert | 5  4  0  0  1  3  4  1  0  0  8  1  0  0  0  5  3  1  0  6  3 | 22  13  0  1  12  13  5  1  3  3  4  18  7  1  2  18  15  2  0  25  10 | 6  4  9  1  0  0  0  0  0  0  0  0  0  10  0  -  -  -  -  -  - | 12  14  26  0  0  0  0  0  0  0  0  0  0  26  0  -  -  -  -  -  - |

Table B- The relevant documents

| **Type of document** | **Title** | **Provider** | **Publication Year** |
| --- | --- | --- | --- |
| Formal reports and programs (9) | 1. Second National Strategic Plan for HIV Control of the Islamic Republic of Iran | National Committee for AIDS Control | - |
|  | 2. Third National Strategic Plan for HIV Control of the Islamic Republic of Iran 2010-2014 | National Committee for AIDS Control | 2010 |
|  | 3. Fourth National Strategic Plan for HIV Control of the Islamic Republic of Iran 2015-2019 | National Committee for AIDS Control | 2015 |
|  | 4. AIDS Monitoring Report in the Islamic Republic of Iran (on monitoring the Declaration of Commitment adopted by the UN General Assembly on HIV and AIDS) | Secretariat of the National AIDS Working Group (Ministry of Health and Medical Education) | 2017 |
|  | 5. Report of the Islamic Republic of Iran (On the Monitoring of the Declaration of Commitment adopted by the Special Session of the United Nations General Assembly on HIV and AIDS) | Secretariat of the National AIDS Working Group (Ministry of Health and Medical Education) | 2009 |
|  | 6. Progress Report on AIDS Control in the Islamic Republic of Iran (on monitoring the Declaration of Commitment adopted by the UN General Assembly on HIV and AIDS) | Secretariat of the National AIDS Working Group (Ministry of Health and Medical Education) | 2014 |
|  | 7. Adolescent and Youth Health and Counseling Club Protocol | Disease Control Center (Ministry of Health Medical Education) | 2016 |
|  | 8. HIV/AIDS program | WHO, UNODC, and UNAIDS | 2012 |
|  | 9. International Drug Use Prevention Standards | UNODC, Drug Control Headquarters of the Islamic Republic of Iran | 2013 |
| Book (4) | 10. A Practical Guide to Life Skills for Youth - Effective Communication Skills (Vol. II) | Mental Health Center, Department of Health and Treatment of Martyr and Veterans Affairs Foundation | 2007 |
|  | 11. AIDS is not just for "others"! (Guidelines on HIV and AIDS for the mass media in the Islamic Republic of Iran) | Center for Disease Control (Ministry of Health and Medical Education) | 2009 |
|  | 12. HIV/AIDS Prevention (Basij Salamat Volunteers Training Guide - Health Volunteers Network - Healthy Lifestyle) | Health Education and Promotion Office of the Department of Health, in cooperation with the Islamic Revolutionary Guard Corps and the Basij Mostazafin Organization and Sabzevar University of Medical Sciences | 2016 |
|  | 13. NGO Guide | Author: Hillary Banderville - Compiled and translated by Mustafa Jamali | 2017 |
| Guide (4) | 14. Training guide to healthy lifestyle for adolescents for trainers and parents/teachers school committee to train parents | In collaboration with the Central Organization of the Parents and Teachers Association, the Department of Health of Shahid Beheshti University of Medical Sciences, the Office of Adolescent and School Health of the Ministry of Health and Medical Education, the UN Population Fund | 2020 |
|  | 15. HIV/AIDS Training Guide (for health trainers) | Center for Disease Control of the Ministry of Health and Medical Education - AIDS and Sexually Transmitted Infections Control Center | 2014 |
|  | 16. A Guide to the New Approach to HIV Education (Public Education Trainers) | Center for Disease Control of the Ministry of Health and Medical Education | 2016 |
|  | 17. Technical, specialized and managerial guide in the field of mental health and addiction, for mental health and behavior experts in health centers and complexes | Department of Health, Tabriz University of Medical Sciences (Department of Mental Health and Addiction Experts) | 2016 |
| Instructions (2) | 18. World AIDS Day Commemoration Rules for Universities / Schools of Medical Sciences and Health Services | Center for Diseases Control, Department of AIDS and Sexually Transmitted Infections - Ministry of Health and Medical Education | 2008 |
|  | 19. Executive instructions and decree for establishing and managing welfare service centers (+ life) | National Welfare Organization | 2020 |
| Rules and regulations (9) | 20. Regulation of the Supreme Planning Council for the Prevention of HIV and AIDS and its Control | Ministry of Health and Medical Education | 2003 |
|  | 21. Regulation of the implementation of activities and responsibilities under the quintet provincial committees for HIV/AIDS prevention and control | CDC, Approved by the National Committee for Epidemiological Care, Monitoring and Evaluation | Winter  2008 |
|  | 22. Regulations for the formation of the Technical Committee for AIDS Social Support | Ministry of Cooperatives, Labor and Social Welfare | 2012 |
|  | 23. Executive regulations of authorized centers for treatment and reduction of harm of drug addiction and psychotropic substances | Ministry of Health and Medical Education, Ministry of Cooperatives, Labor and Social Welfare, Ministry of Interior, Secretary General of Drug Control Headquarter | 2013 |
|  | 24. Regulations of non-governmental organizations | Social Welfare Organization | 2016 |
|  | 25. Law on organizing street children | Social Welfare Organization | 2019 |
|  | 26. Organization and duties of the AIDS Prevention and Control Committee | Social Welfare Organization | - |
|  | 27. Regulations for establishing a service center to reduce substance dependence | Ministry of Health and Medical Education | - |
|  | 28. Regulations of the High Council for Health and Food Security | The Planning and Budget Organization, the Ministry of Jihad, and the Ministry of Health jointly proposed this regulation based on paragraph (b) of Article 7 of the Law on Permanent Provisions of the Country's Development Plans, approved in 2016. | 2018 |
